# A Randomised Controlled Feasibility Trial of Immersive Virtual Reality Treatment with Cognitive Behaviour Therapy for Specific Phobias in Young People with Autism Spectrum Disorder

**DOI:** 10.1007/s10803-018-3861-x

**Published:** 2019-02-15

**Authors:** Morag Maskey, Jacqui Rodgers, Victoria Grahame, Magdalena Glod, Emma Honey, Julia Kinnear, Marie Labus, Jenny Milne, Dimitrios Minos, Helen McConachie, Jeremy R. Parr

**Affiliations:** 1 Institute of Neuroscience, Sir James Spence Institute Level 3, Royal Victoria Infirmary, Newcastle University, Newcastle upon Tyne, NE1 4LP UK; 2 Complex Neurodevelopmental Disorders Service, Northumberland Tyne and Wear NHS Foundation Trust, Newcastle upon Tyne, UK; 3 Newcastle University, Newcastle upon Tyne, UK; 4 Business Development and Enterprise, Faculty of Medical Sciences, Newcastle University, Newcastle upon Tyne, UK; 5 Tees Esk and Wear Valley NHS Foundation, Trust, UK; 6 Institute of Health and Society, Newcastle University, Newcastle upon Tyne, UK

**Keywords:** Autism, Anxiety, Phobia, Fear, Virtual reality, Cognitive behaviour therapy

## Abstract

We examined the feasibility and acceptability of using an immersive virtual reality environment (VRE) alongside cognitive behaviour therapy (CBT) for young people with autism experiencing specific phobia. Thirty-two participants were randomised to treatment or control. Treatment involved one session introducing CBT techniques and four VRE sessions, delivered by local clinical therapists. Change in target behaviour was independently rated. Two weeks after treatment, four treatment participants (25%) and no control participants were responders; at 6 months after treatment, six (38%) treatment and no control participants were responders. At 6 months post-treatment, symptoms had worsened for one treatment and five control (untreated) participants. Brief VRE exposure with CBT is feasible and acceptable to deliver through child clinical services and is effective for some participants.

## Introduction

Autism spectrum disorder (ASD) occurs in around 1% of the population and is characterised by social communication difficulties and repetitive behaviours (American Psychiatric Association 2013; Baird et al. 2006). Co-existing conditions are common in ASD (Maskey et al. 2013) including anxiety, which affects around half of children (Simonoff et al. 2008; Maskey et al. 2013). In a clinical setting, anxiety is among the most common treatment referral reasons for young people with ASD (Ghaziuddin et al. 2002). Specific phobia (defined by DSM-5 as extreme or irrational fear of an object/situation) is one of the most common anxiety presentations in ASD (Leyfer et al. 2006; Mattila et al. 2010). Prevalence rates of 30–64% have been reported (Leyfer et al. 2006; van Steensel et al. 2011); rates are 5–18% in typically developing children (Ollendick et al. 2002). The nature of phobias for children with ASD may be ‘unusual’ or atypical, such as situation-specific fear (e.g. visiting a particular location), of everyday objects (toilets, machines, foods), or of people with certain personal characteristics (Mayes et al. 2013). Importantly these difficulties have an impact on the daily lives of the child and family, interfering with education and learning (Maskey et al. 2014) and have been associated with higher levels of challenging behaviours (Evans et al. 2005).

There are a range of treatments for phobias in typically developing individuals, with cognitive behaviour therapy (CBT) and graded exposure as key therapeutic techniques (Ollendick et al. 2006). However, these techniques may not be as effective for some children with ASD. For example, graded exposure typically begins with imaginal desensitisation; many individuals with ASD experience difficulties with imagination (Lind et al. 2014) such as producing and controlling imaginal scenes. This may be a challenge or barrier to treatment adherence and/or effectiveness. National Institute for Health and Care Excellence (NICE) ASD management guidance in the UK specifies that CBT will require adaptation to increase the likelihood of effectiveness for individuals with ASD (NICE 2013). Suggested adaptations include the development of disorder-specific hierarchies, the use of more concrete visual tactics, incorporation of a child’s specific interests, and inclusion of parents in treatment (Moree and Davis 2010); additional adaptations may include psychoeducation about recognising and understanding emotions, problem solving, and a reduced cognitive component with greater use of behavioural strategies such as exposure and relaxation. Research indicates that with such adaptations, CBT based interventions can be successful in promoting anxiety reduction for individuals on the autism spectrum (Lang et al. 2010; Ung et al. 2015).

Increasingly, new technologies, such as virtual reality (VR), are being used with the neurotypical population to augment traditional psychological treatments (Hollis et al. 2016; Freeman et al. 2017; Valmaggia et al. 2016). VR may be particularly helpful for the delivery of interventions for those with ASD because it allows simulations of real world situations to be created, and newly learned coping skills can be rehearsed and reinforced in a safe and controlled environment (Parsons and Cobb 2011). VR has been used successfully to improve various skills, for example, social understanding (Mitchell et al. 2007; Kandalaft et al. 2013), job interview skills (Smith et al. 2014), driving skills (Bian et al. 2013) and road safety and fire alarm procedures (Josman et al. 2008; Strickland et al. 2007). For people with ASD and specific phobias, VR may offer an alternative to usual exposure hierarchies used in traditional CBT, which typically move through imaginal desensitisation to real life exposure. VR may facilitate a more gradual exposure to the feared stimulus in a controlled manner, whilst concurrently allowing the participant to be supported to develop anxiety management strategies with a therapist.

Given the potential utility of the combination of CBT and VR to the treatment of specific phobias for people with ASD, we aimed to investigate the combination of an immersive virtual reality environment (VRE) alongside therapist-delivered CBT to reduce anxiety. Maskey et.al. (2014), using a case study design with nine volunteer children who experienced specific phobias, reported the preliminary acceptability of an immersive VRE, known as the Blue Room, alongside CBT. The Blue Room is a fully immersive VRE without the need for a headset or goggles; the therapist controls the perceived movement through the scene with an iPad. Each child received four VRE twenty-minute treatment sessions delivered over one week. Of nine volunteer children, eight children improved in their ability to tackle their real life specific phobia, as described in vignettes of behaviour and rated by an expert panel. These improvements were maintained at 12 months follow up. The specific phobias addressed were related to everyday occurrences, such as travelling on public transport, and therefore overcoming these phobias significantly improved families’ daily lives (Maskey et al. 2014). Limitations included recruitment from a community based group rather than from clinical health services, lack of randomisation and a control group, and treatment delivery by the study team rather than within families’ local health services.

In order to provide further evidence, we aimed to trial the VRE and CBT intervention reported in Maskey et al (2014) with a larger sample of children who were being seen in clinical services, randomising them to either a treatment or control group (delayed treatment). We report the results of the first Randomised Controlled Trial (RCT) of the Blue Room VRE intervention. The study aims were to (1) evaluate treatment delivery feasibility, with fidelity, by therapists from two UK National Health Service (NHS) teams; (2) determine acceptability of outcome measures to young people and parents; (3) investigate responses to the VRE treatment; (4) monitor whether initial benefits from treatment persisted.

## Methods

### Study Design

This was a single blind RCT comparing a virtual reality treatment for specific phobias in children with ASD with usual care (immediate and control (delayed) treatment arms). After consent and baseline measures were taken, participants were randomised to one of these arms.

The immediate treatment group were followed up at 2 weeks and 6 months after treatment. The control arm completed outcome measures at these time points. The control group then received the treatment after completing the measures at 6 months (see Consort Diagram); after their treatment they were subsequently followed-up at 2 weeks and 6 months post treatment. The immediate treatment group had one additional follow-up at 12 months post treatment.

### Participants and Recruitment

Thirty-two young people with ASD were recruited over a 12 month period from two UK mental health services—Northumberland, Tyne and Wear NHS Foundation Trust and Tees, Esk and Wear Valley NHS Foundation Trust.

Inclusion criteria: Age 8–14 years, diagnosis of ASD, verbally fluent and able to understand instructions to enable treatment participation and completion of outcome measures. Verbal fluency and comprehension were determined as part of usual clinical practice by the child’s clinical consultant, who judged the child’s suitability for the study. All participants had a specific phobia as identified during the Anxiety Diagnostic Interview Schedule (ADIS) interview (see below). Exclusion criteria were participants whose phobia could not be visually represented and addressed in the Blue Room (e.g. phobia of injections), children with severe and complex anxiety disorder, and/or children with a learning disability that the referring mental health clinician judged would affect their ability to participate.

The child’s usual mental health clinician initially discussed the study with the family and then completed an expression of interest form and the Children’s Global Assessment Scale score (CGAS—a brief measure of emotional and behavioural functioning, with range 1–100) (Shaffer et al. 1983). All participants had a confirmed ASD diagnosis according to DSM-IV or ICD-10 criteria from NHS multidisciplinary diagnostic teams.

On receiving the Expression of Interest form, a member of the research team contacted families to evaluate whether their specific phobia was suitable for treatment and for computer generated, graded scene design. Forty families were identified by clinicians; five of these did not have a phobia that could be treated in the Blue Room and three declined to take part after receiving further information. Thirty-two families received a preparatory home visit where baseline measures were completed and the family shown a video of the Blue Room VRE; written informed consent was obtained. Table 1 shows the characteristics of participants. The two groups were well matched for mean age and gender and on outcomes captured by the standardised measures. Table 2 shows the specific phobias that were identified by participants and their families as the target to address during the Blue Room treatment.

**Table 1:** Baseline characteristics of the immediate treatment and control groups

	Immediate treatment N (%)	Control group N (%)	All children N (%)
Gender			
Male	13 (81.3)	12 (75)	25 (78.1)
Female	3 (18.8)	4 (25)	7 (21.9)
Age			
Mean (months)	130.13 (28.38)	129.00 (21.51)	129.56 (24.78)
Range (months)	89–174	90–157	89–174
Ethnicity			
White	16 (100)	14 (87.5)	30 (93.8)
Non white		2 (12.5)	2 (6.2)
Additional diagnoses			
Any	13 (81.3)	11 (68.8)	24 (67)
Dyslexia	1 (6.3)	1 (6.3)	2 (6.3)
Dyspraxia	3 (18.8)	4 (25.0)	7 (21.9)
ADHD	4 (25.0)	4 (25.0)	8 (25.0)
Other	5 (31.3)	2 (12.5)	7 (21.9)
Household income			
Above UK mean income	9 (56.3)	9 (56.3)	18 (56.3)
Below UK mean income	6 (37.5)	7 (43.8)	13 (40.6)
Prefer not to say	1 (6.3)	0	1 (3.1)
Information about parent who observed treatment			
Mother/Father (includes one grandmother)	15/1 (93.8/6.3)	14/2 (87.5/12.5)	29/3 (90.6/9.4)
Married/cohabiting	12 (75.0)	13 (81.3)	25 (78.1)
University degree	6 (37.5)	3 (18.8)	9 (28.1)
Employed	10 (62.5)	9 (56.3)	19 (59.4)
SCQ^a^ score ≥ 15/<15	14/1	14/2	29/3
Mean SCQ score (SD)	25.07 (7.69) (1 missing)	25.06 (7.59)	25.06 (7.51) (1 missing)
Mean CGAS^b^ score (SD)	52 (13.7) (9 participants)	49 (7.7) (7 participants)	50.81 (11.26) (16 participants)
ADIS^c^ primary diagnosis			
Specific phobia	14 (87.5)	14 (87.5)	28 (87.5)
Social phobia	2 (12.5)	2 (12.5)	4 (12.5)
Number with secondary diagnoses (mean number of secondary diagnoses per child)	14 (2.8)	14 (2.8)	28 (2.8)
Mean Vineland scores	(n = 14)	(n = 16)	(n = 30)
Communication	73.50 (16.09)	73.63 (11.91)	73.57 (13.76)
Daily living skills	72.07 (13.68)	65.44 (8.10)	68.57 (11.33)
Socialisation	63.14 (10.38)	65.00 (14.97)	64.40 (12.27)
Adaptive behaviour composite	68.43 (11.99)	66.63 (8.53)	67.53 (10.08)

^a^*SCQ* Social Communication Questionnaire

^b^*CGAS* Children’s Global Assessment Scale

^c^*ADIS* Anxiety Disorders Interview Schedule

**Table 2:** Specific phobias which were addressed (treatment group and control group)

Treatment group phobias	Control group phobias
Bananas	Dogs (x2)
Wasps/bees (x2)	Flying (x2)
Open spaces	Wasps/bees
Dogs (x3)	Specific chronological time
Lifts	Heights/glass elevators (x2)
Fear of the dark	Thunder and lightening
Insects	Making requests^a^
Being looked at^a^	Mascots
Changes in weather	Automated toys
Eating in front of other people^a^	Fear of the dark
Balloons	Travelling in the car
Dolls	Toilets
Bats	Balloons

^a^Anxiety related to very specific social situations that were identified by the child and their parents as highly desirable treatment targets

### Measures

#### Baseline Characterisation

The following measures were completed by the participant and their parent/caregiver at baseline.

##### Social Communication Questionnaire (SCQ) (Berument et al. 1999)

A parent-completed 40 item questionnaire to describe the child’s ASD characteristics. It is used internationally, and has high sensitivity and specificity for an ASD diagnosis.

##### Anxiety Disorders Interview Schedule (ADIS) (Silverman 1996)

A widely used standardised clinical interview carried out with parents; each anxiety area is given a severity rating, including separation anxiety, social anxiety disorder, specific phobia, panic disorder, obsessive–compulsive disorder and generalised anxiety disorder.

##### Vineland Adaptive Behaviour Scales (VABS) (Sparrow et al. 2005)

This parent interview allows children’s functional abilities to be compared to age norms (Communication, Socialisation and Daily Living Skills). We did not undertake IQ assessment as feasibility must relate to current clinical practice, where clinicians take pragmatic decisions about treatment and children’s capabilities rather than basing treatment access on assessment scores.

### Outcome Measures

Maskey et al (2014) reported that Target Behaviour ratings are appropriate outcome measures of real life change in behaviours of concern. This is further supported in a review of the treatment of specific fears and phobias in children with ASD (Lydon et al. 2015), where target behaviours were found to be the primary outcome measure in 10 of the 16 studies reviewed. This measure was therefore included as the main outcome measure, alongside questionnaires, to investigate the utility of different outcome measures with this clinical population.

For the treatment and control groups the following outcome measures were collected:

*Target Behaviour rating* This measure recorded a rating of change over time in the specific phobia to be tackled through Blue Room treatment. The protocol was developed by the Research Units on Paediatric Psychopharmacology (RUPP) Autism Network (Arnold et al. 2003). Questions regarding the child’s specific phobia behaviours, and questions such as ‘how often?’ and ‘how distressed?’ were asked in a standard format to the parent, enabling a vignette to be written. Following an interview about phobia during the initial home visit, pre-treatment vignettes were written prior to randomisation, by the first author (for examples see Table 5). These baseline vignettes are not rated per se, but rather serve as a baseline from which Target Behaviour change is measured. Follow-up vignettes were written from telephone interviews with parents, undertaken by the blinded outcome assessor. All efforts were made to maintain the assessor as blinded. Each vignette pair (baseline vs post treatment vignettes) was evaluated for *change* over time on a 9 point scale (from ‘normalised’ to ‘disastrously worse’). An expert panel of raters received training with examples, before rating the pairs of vignettes. Raters were blind to group allocation and time point, and each vignette pair was rated by four different raters. Arnold and colleagues (Arnold et al. 2003) reported an Intraclass Correlation Coefficient (ICC) of 0.895 for a panel of 5 experts; in this study ICCs for the two time points were 0.869 (95% CI 0.775 to 0.930) and 0.935 (95% CI 0.887 to 0.965). The Target Behaviour rating is reported both dimensionally, and also by categorical cut-score using a mean of 3.0 or less, corresponding to a rating of ‘definitely improved’ or better, to define positive treatment response (‘responders’). In addition, a cut-score of 6.0 or more was used in this study to define those whose symptoms had worsened compared to baseline.

*Spence Children’s Anxiety Scale-parent version* (*SCAS-P*) *and child version* (*SCAS-C*) The SCAS (Spence 1998) was developed to assess anxiety symptoms in children in the general population and has 38 items with a 0 (never) to 3 (always) scale. The measure has been widely used in ASD studies (Sofronoff et al. 2005; Maskey et al. 2014). High internal consistency for the total scale score has been reported (Spence 1998), and both convergent and divergent validity (Nauta et al. 2004). In the current study, internal consistency at baseline was α = 0.900 for SCAS-P and α = 0.863 for SCAS-C.

*Fear survey schedule for children—revised* (*FSSC-R*) (Ollendick 1983) This is an 80 item parent-report questionnaire with an overall intensity and fearfulness score. The FSSC-R is the most commonly used tool for assessment of common fears and phobias, with good construct, convergent and divergent validity (Gullone et al. 2000) and strong test–retest reliability and internal consistency (Burnham and Gullone 1997). In the current study, internal consistency at baseline was α = 0.932.

*Children’s Assessment of Participation and Enjoyment* (*CAPE*). This was completed by the child at baseline and 9 months and intended to measure any increase in participation in community activities. CAPE is a 50 item child-report of activities, presented pictorially, to assess children’s participation in a range of solitary and group voluntary activities. Reliability and validity of the CAPE was established through study of 427 children with disabilities (King et al. 2007).

### Process Measures

Attendance: whether children attended all sessions was recorded.

Confidence ratings: during treatment children rated their confidence at tackling their goal situation at the beginning of session one, end of session two, beginning of session three and end of session four. Parents rated their perception of their child’s confidence at parallel time points. Ratings were from 0 (not comfortable) to 6 (very comfortable); parent and child ratings were taken in separate rooms and not shared. Examples of a confidence scale used are given in a previous publication (Maskey et al. 2014).

When families were approached but chose not to participate, ethical permission was granted to pass the following anonymised data to the research team: CGAS score, age, gender, ASD diagnosis and type of diagnosis. This was to allow ‘refusers’ to be characterised and compared with trial participants.

### Randomisation and Masking

Participants were randomly allocated to immediate treatment group (n = 16) or control group, for whom treatment was offered after the 6 months outcome measures were administered (n = 16). Allocation was by computer using a password-protected Newcastle University Clinical Trials Unit website. Randomisation was by mixed block design, using block sizes of two and four, stratified by site. Due to the nature of the treatment, participants, clinicians and the main researcher for the study were aware of group allocation. Participants were randomised and informed after the initial home visit as to whether or not they were in the immediate treatment group. Another researcher, blind to treatment allocation, conducted outcome measurements through telephone discussion of target behaviours with families and receiving postal questionnaires. Blinding was strictly maintained; this outcome assessor had no other trial role, no access to documents and did not attend trial meetings. At each telephone or postal contact, this outcome assessor reminded parents she was unaware of group allocation.

### Materials

**Fig. 1:**
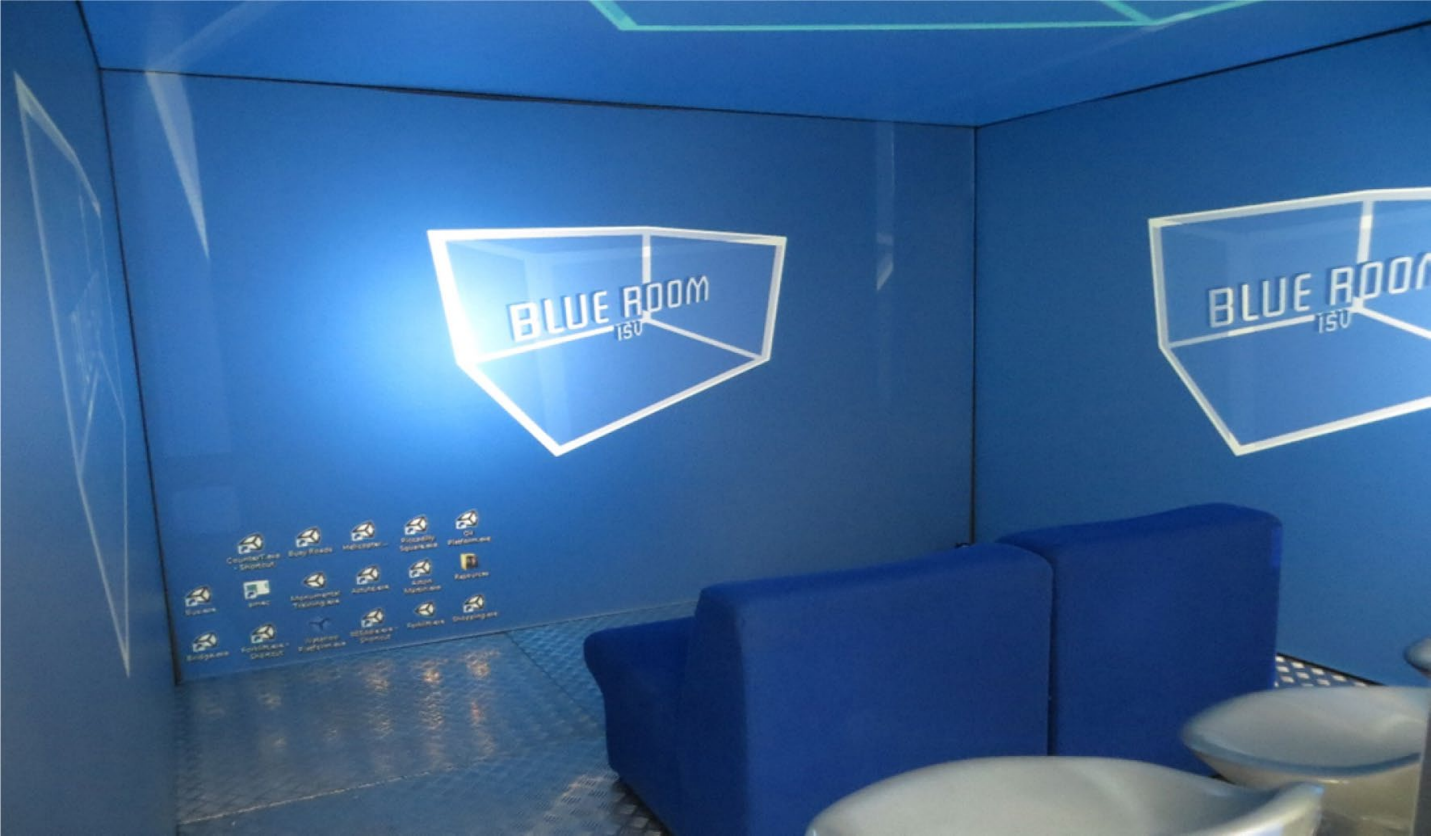
Picture of the Blue Room virtual reality environment

The Blue Room VRE is a patented immersive technology using interactive computer generated audio visual images projected onto the walls and ceilings of a 360 degree screened room (Fig. 1). The room was 4 m^3^ and the participant and therapist sit side by side. A therapist remains with the participant throughout the treatment sessions, delivering the CBT techniques (described below). Scenes are individualised, incorporating an exposure hierarchy related to the feared stimulus. For example, for dog phobia, adaptions include the dog’s size, whether on or off a lead, barking, and proximity to the participant. This gradation allows the participant to experience levels of mastery in managing their anxiety and to repeatedly practice this at one level of challenge before moving to the next (Maskey et al. 2014). The following link shows a session in progress: https://www.youtube.com/watch?v=9U-rRC8jc28.

### Treatment

Before VR sessions, each participant and parent attended a 45 minutes session with their allocated therapist. The therapist was a health professional (for example an assistant psychologist, or a specialist nurse) with experience in ASD and/or CBT, who had attended the training workshop (see below). Simplified CBT techniques were introduced, including: (1) identifying feelings (how different parts of the body feel; how thoughts, emotions and behaviours are connected); (2) the concept of a visual ‘feeling thermometer’ using the participant’s words to describe anxiety; (3) two relaxation exercises (muscle relaxation and deep breathing, with scripts for home practice); (4) identification of the participant’s positive coping statement, e.g. ‘I can do this’, ‘I’m going to be ok’, to use in the treatment sessions. These CBT elements were repeated and consolidated during VRE sessions. The goal for the end of treatment was agreed with the participant; this goal was used for the confidence rating charts for parent and participant.

Following scene creation, participants attended 20 min treatment sessions. Two Blue Room treatment sessions were completed at one visit, with a fifteen minute break between. The second two sessions were conducted around one week later. The therapist allocated to the participant was present during all sessions. Parents watched treatment via a video link, and the session content and purpose of activities was explained. For the first two sessions, a supervising qualified clinical psychologist attended to observe and give feedback to the therapist. This supervising therapist also answered any questions the family had during the session.

Materials for the treatment sessions (the treatment manual, customised visual scales and relaxation scripts) were provided to therapists. Each Blue Room session started with a relaxation scene, allowing the participants to become familiar with the environment, and to practice relaxation techniques and coping self-statements. The two available relaxation scenes were of swimming dolphins, and a field in the country; scenes had soft background music that could be turned off if requested. The duration the child spent looking at these scenes and practising relaxation exercises was at the discretion of the therapist, as the aim was to be responsive to the needs of individual participants; for most participants, one cycle of muscle relaxation and breathing exercises was sufficient at the beginning of each session. The relaxation scenes were returned to during a session if the therapist thought the participant’s anxiety was severe or if the participant was finding it difficult to manage their anxiety during a particular scene.

Following the relaxation scenes, the participant was introduced to the VRE scene designed for them. The initial scene was designed to be the lowest level of the exposure hierarchy for that participant (e.g. a quiet dog on a lead in the distance). The participant gradually moved through the hierarchy; they progressed to an increased level of challenge when they were consistently reporting low levels of anxiety (score of 2 or less on a six point scale) on the visual scale for a scene, and there was agreement between the participant and therapist to move on. At each level of the hierarchy, participants were supported by the therapist to practise techniques to reduce anxiety, including relaxation exercises, thought challenging and anxiety monitoring. If anxiety increased as the scene became more challenging, the therapist suggested relaxation and breathing exercises, moving to the relaxation scenes if needed. Progress through the scenes was determined by the progress participants made towards maintaining low anxiety at each level and was individualised to each participant. After completing the fourth session, the therapist spoke with participants and their family regarding graded real world exposure to the anxiety situation. The therapist explained the need to gradually introduce the participant to the feared situation in real life and discussed various steps in the hierarchy of exposure relevant to the particular phobia.

### Therapist Training and Treatment Fidelity Measurement

Before delivering treatment, local therapists read the manual and attended a 2 hours, group training workshop delivered by an experienced child clinical psychologist (author 10). The manual for the treatment is copyrighted and is available on request from the corresponding author. Training involved discussion of why children with ASD may develop anxiety, explanation of the steps in treatment including evolution over four sessions, review of video material from live sessions, and individual practice with the tablet computer in the VRE.

All Blue Room treatment sessions were video recorded. A sample of 30% of sessions in the immediate treatment group were rated for fidelity to delivery as per the manual. The sessions were chosen at random but always included at least one session conducted by each of the 11 therapists, and an even spread of VRE sessions 1 to 4. Fidelity was recorded on a checklist to assess (a) Delivery of CBT best practice and (b) the manual Content and Structure [checklist designed by author 2, drawing on sources including Roth & Pilling (Roth and Pilling 2008)]. Delivery ratings included Techniques used (9 or 10 elements e.g. collaborative approach, modelling reflection, using relaxation strategies, using praise), Generic Acceptable components (5 elements e.g. therapeutic alliance, managing emotional content, appropriate flexibility) and Undesirable components (6 elements e.g. didactic approach, allows off-topic deviation); Content and Structure included around 10 elements (e.g. setting agenda, summarising, scenes presented in increasing levels of difficulty). Rating definitions and number varied between sessions for Techniques and Content, as different elements were introduced or became irrelevant. Delivery ratings were: 0 (not at all), 1 (minimal evidence), 2 (several examples) with ratings reversed for Undesirable components. Content ratings were: 0 (not covered), 1 (covered insufficiently) and 2 (covered adequately). Senior co-authors (authors 2, 3 and 10) established mean inter-rater agreement at 83.6% for Techniques, 96.0% for Acceptable and 92.2% for Undesirable components. Agreement of mean 69.7% (range across raters and sessions 56–94%) for Content and Structure was lower so was not rated further. Content rating proved difficult for several reasons: the quality of audio in recordings; some aspects perhaps being covered outside the VR; expectation that CBT would be flexibly individualised.

### Analysis

Analysis was conducted according to a pre-specified statistical analysis plan. Post hoc testing of the main outcome measure found sensitive to change in our development study (Target Behaviour rating) was then conducted to explore potential efficacy.

Analysis was undertaken by author 4, blind to group status and supervised by author 2.

Group equivalence at baseline was investigated using Fisher’s exact test, Pearson’s chi square and t-tests. Exploratory group comparison over time was made for the Target Behaviour ratings using Mann Whitney U test and chi square. Descriptive statistics (mean, standard deviation) are presented for all questionnaire data, along with effect size (Cohen’s d), where values 0.2, 0.5 and 0.8 indicate a small, medium, and large effect size respectively.

Collection of 12-month post treatment data from the treatment group: To undertake a preliminary investigation of the medium term treatment effectiveness, children’s outcomes for the immediate treatment group were investigated 12 months post treatment. Target behaviour follow-up vignettes were written following telephone interviews with parents; these were undertaken by the same blinded outcome assessor and vignettes written (questionnaire data were not collected at this timepoint to reduce the burden on parents). Vignettes were rated as described above by the blinded expert rater group. Descriptive data are reported separately from the main trial outcomes.

Treatment outcomes for the control (delayed treatment) group: After the wait phase and completion of measures at 6 months, parents and children from the control group were offered VR treatment sessions. 15/16 parents accepted; one child was not treated as following discussion with the local treating clinician, it was agreed that due to family circumstances and generalised anxiety symptoms, phobia treatment was not the main priority. Fifteen children received treatment according to the treatment plan described above. Outcome measures were collected at 2 weeks and 6 months post treatment, and target behaviour interviews were undertaken with parents; vignettes were written by the same outcome assessor, who at this stage was not blind as only control group data was being collected at this point. Questionnaire data were not collected. Vignettes were rated as described above by the expert rater group, who remained blind to group status. Descriptive data are reported separately from the main trial outcomes.

### Approvals and Research Governance

A favourable ethical opinion was provided by the UK National Health Service Tyne and Wear South Ethics Committee (reference 14/NE/1177). The research sponsor was Northumberland, Tyne and Wear NHS Foundation Trust. The trial was conducted and reported in accordance with CONSORT guidelines, and was registered on the ISRCTN trial database (Trial ID: ISRCTN7886185). The trial team and co-applicants met regularly during the study. A parent advisory group met regularly to advise on trial procedures.

## Results

Participants were recruited between March 2015 and February 2016. Thirty-two children, mean age 10 years 10 months, were recruited (25 boys and 7 girls); 16 children were allocated to each trial arm. The baseline characteristics of children from the immediate treatment and control groups are shown in Table 1. The two groups did not differ significantly on any measure of child and family demographics, the presence of additional diagnoses that commonly co-exist with ASD, nor baseline characterisation measures, i.e. children’s ASD symptom severity, anxiety diagnoses, level of adaptive behaviour, and overall functioning.

Some information was available from the children with ASD who were approached and chose not to participate or who did not have a suitable phobia to participate in the trial. This included 7 boys and 1 girl (mean age 11 years 3 months). Three CGAS scores were available; the mean CGAS score for non-participants was 45.0 (SD 9.6), similar to the mean score of the total sample.

### Feasibility and Acceptability

**Fig. 2:**
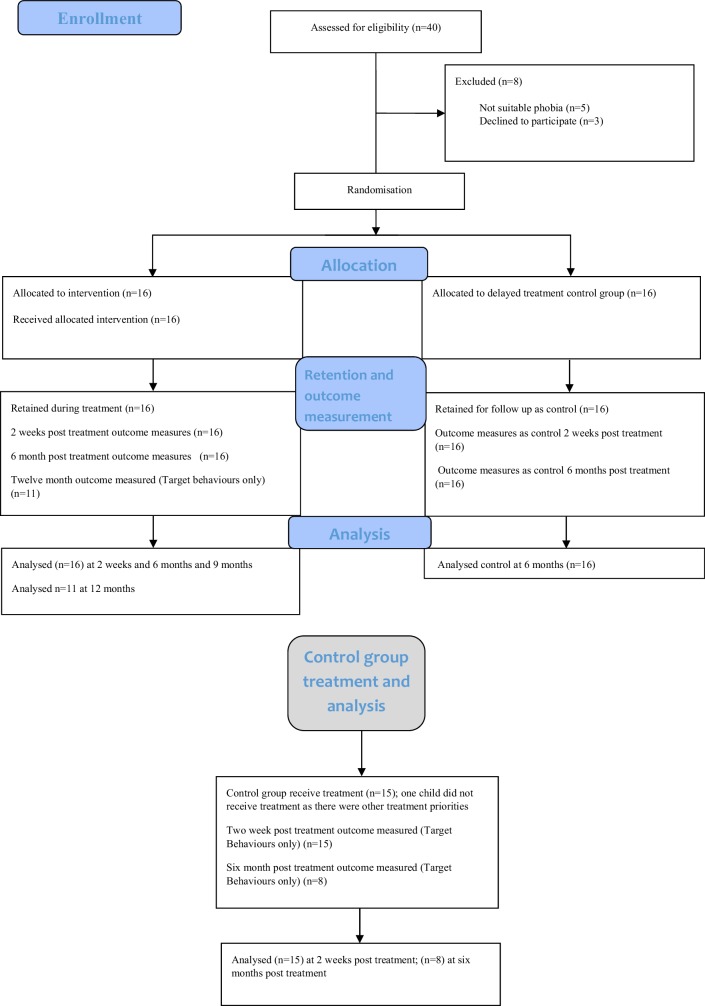
Consort diagram

Figure 2 shows the flow of participants through the trial. All 32 participants completed the interview about the target behaviour at all time points. There was no attrition by six months. Data completeness for questionnaires was 89.0% at baseline and 82.8% at 6 months follow-up.

All children in the immediate treatment group completed four VRE treatment sessions.

**Fig. 3:**
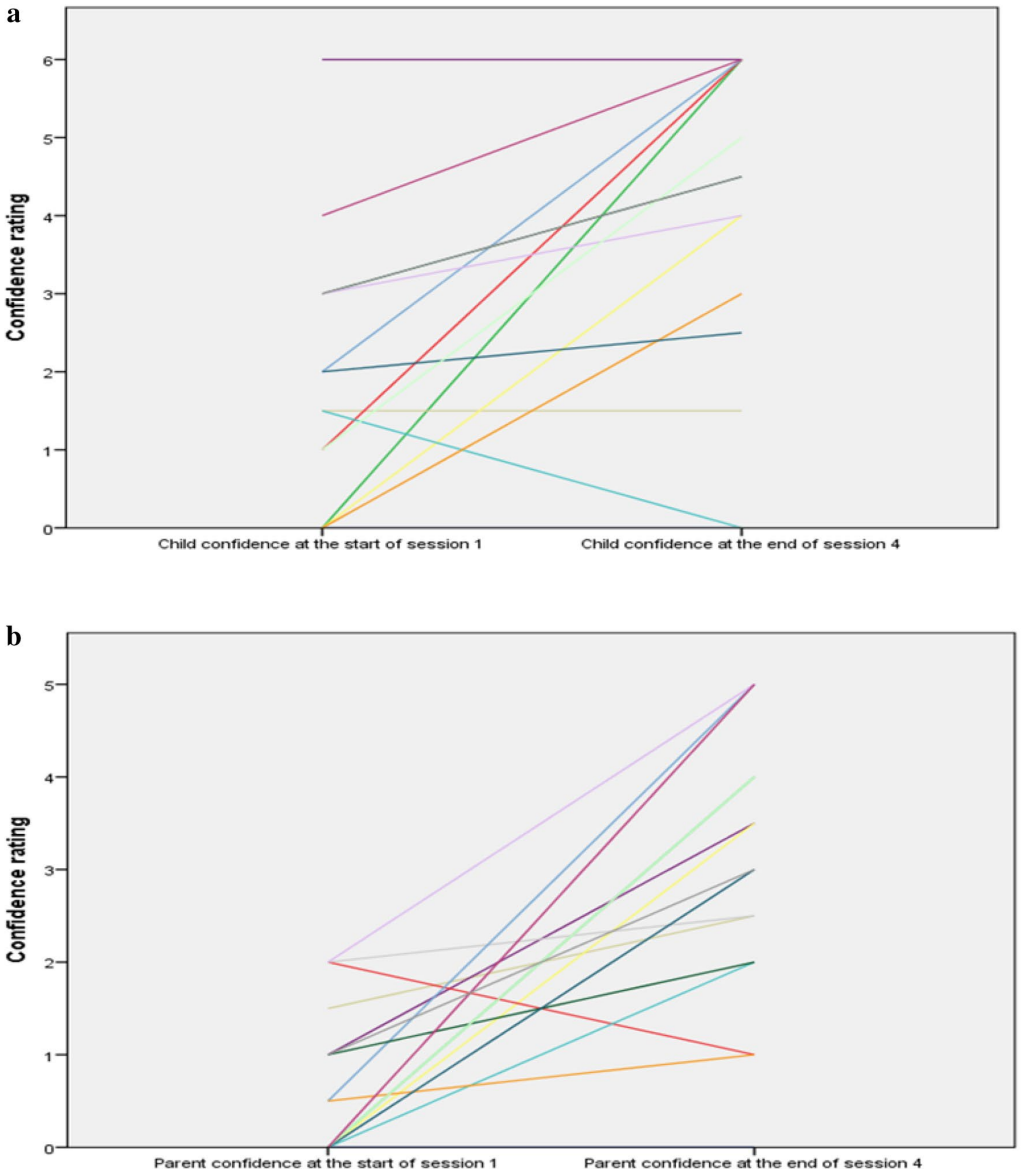
**a** Change in child ratings of their own confidence from treatment session 1 to 4 for the immediate treatment group. **b** Change in parent’s rating of their child’s confidence from treatment session 1 to 4 for the immediate treatment group

For the immediate treatment group, most child and parent confidence ratings for tackling the goal situation increased between the start of session 1 and at the end of session 4 (Fig. 3a, b). The average fidelity of treatment delivery across sessions was very high at 94.5% (range 91–96.5%). The overall mean fidelity for the eleven therapists ranged from 84.5-100% indicating all therapists were at least adequate and most were excellent in style of delivery of the VRE CBT treatment.

### Treatment Outcomes

#### Target Behaviour Rating

The treatment group showed a statistically significant greater improvement on Target Behaviour ratings compared with the control group, for both baseline to two weeks post treatment (U = 67.5, p = 0.021) and baseline to six months post treatment (U = 53.0, p = 0.007) (Table 3) with large effect sizes.

**Table 3:** Target behaviour mean change ratings for immediate treatment and control groups at 2 weeks and at 6 months post treatment with effect size and observed power. Lower score indicates greater improvement

	Treatment		Control		Total		Effect size Cohen’s *d*	95% CI for Cohen’s *d*		Observed power
	n	Mean	n	Mean	n	Mean		Lower	Upper	
Rating at 2 weeks	16	4.17 (1.27)	16	5.25 (0.77)	32	4.71 (1.17)	1.03	0.291	1.766	.803
Rating at 6 months	16	3.92 (1.63)	15	5.40 (0.86)	31	4.64 (1.50)	1.14	0.377	1.894	.852

Six out of 16 (38%) treatment group children were classified as responders six months after treatment, compared with no control group children (responders vs all others, χ^2^ = 6.98, p = 0.018) (Table 4). Four children in the treatment group had responded at 2 weeks post treatment. It is important to note that, at 6 months, five children from the control group (31%) were rated as having symptoms worse than baseline; one of the immediate treatment group also showed symptoms worsening. Twelve months post treatment data for the immediate treatment group were available from 11/15 children; four of these 11 children were treatment responders. Table 5 shows examples from edited vignettes at baseline, and 6 months post treatment, for three exemplar treatment group children (one responder, one no/equivocal change and the child whose symptoms were worse).

**Table 4:** Target behaviour ratings categories (responder, no change/equivocal, and worse) for immediate treatment group at 2 weeks, 6 months and 12 months post treatment and control group

Target behaviour rating	2 weeks		6 months		12 months
	Treatment	Control	Treatment	Control	Treatment
	n (%)	n (%)	n (%)	n (%)^a^	n (%)^b^
Treatment responder (1.0–3.0)	4/16 (25.0)	0	6/16 (37.5)	0	4/11 (36.4)
No change/equivocal (3.1–5.9)	11/16 (68.8)	13/16 (81.3)	9/16 (56.3)	10/15 (66.7)	7/11 (63.6)
Symptoms worse than at baseline (6.0–9.0)	1/16 (6.3)	3/16 (18.7)	1/16 (6.3)	5/15 (33.3)	0

^a^1 parent vignette missing

^b^5 parent vignettes missing

**Table 5:** Extracts from exemplar vignettes ratings: responders, no change/equivocal, symptoms worse

Extracts from vignettes		
	Baseline	6 month follow-up
Responder (≤ 3)		
Average Target Behaviour rating 3.00	He has a phobia of dolls. When he sees a doll, he becomes very irritable and wants to get away. His fear reaction lasts as long as the doll is in his sight or on his mind. He cannot eat if he sees a doll. He has vomited on occasion in a restaurant when someone went past with a doll	His phobia is not a huge problem at the moment. He has become very tolerant recently when he is near any dolls. His reaction to seeing dolls only lasts as long as it is front of him. He can spot a doll from very far away
No or equivocal change (3.1–5.9 rating)		
Average rating 5.00	He has a phobia of balloons. If he sees a balloon he will always try to run away. He screams and cries inconsolably. The colour drains from his face and his heart races. Whenever the family goes out, he will begin questioning ‘but what if there’s balloons?’	His phobia causes problems whenever he is in a situation where there may be balloons. He will always ask if there will be any balloons if they are going to visit a new place. If he sees a balloon he will curl up in a ball, cry a lot and his heart will pound very quickly
Symptoms worse (≥ 6)		
Average rating 6.75	He has a phobia of apples and becomes anxious with even a picture of an apple or the word ‘apple’. Every time he sees an apple, or believes someone has been eating an apple on a chair he is about to sit on, he becomes distressed. If he does encounter an apple he becomes agitated, retching for 20 min to one hour. He can run away when out of the house and/or hide behind his mum	He gets very anxious if he thinks there may be apples where he is going. He gets very distressed if he sees someone eating an apple or knows if someone has just eaten one. It is a daily problem and he becomes anxious in relation to apples at least once a day. The reaction lasts between 20 min and 1 h. He will cry, try to run away, and retch if he encounters someone eating an apple. He looks very frightened and his whole body becomes rigid

#### Questionnaire Measures (SCAS-P, SCAS-C, FSSC-R, CAPE)

Questionnaire data (mean total scores, standard deviations and Cohen’s *d* at baseline and 6 months post baseline) for the treatment and control groups are presented in Table 6. None of the comparisons from baseline was statistically significant (p values not shown). To compare groups’ mean questionnaire scores over time mixed factorial ANOVA were performed, with the group (control vs treatment) and time (baseline, 2 weeks post-treatment, 6 months post-treatment) entered as independent variables. All main effects of group and interactions between time and group were non-significant for all the questionnaires’ scores. We present here the 6 month outcome data for ease of reading.

**Table 6:** Mean questionnaire scores at baseline and at 6 months post treatment

	Immediate treatment		Cohen’s *d*	95% CI for Cohen’s *d*		Control		Cohen’s *d*	95% CI for Cohen’s *d*	
	Baseline	6 months		Lower	Upper	Baseline	6 months		Lower	Upper
FSSC-R^a^										
n	15	14				14	12			
Total fearfulness	161.07 (22.46)	153.14 (29.02)	0.3	− 0.427	1.038	155.86 (28.91)	140.17 (22.43)	0.6	− 0.18	1.393
Intense fears	26.13 (12.18)	21.07 (13.34)	0.4	− 0.339	1.131	22.00 (15.33)	18.33 (10.77)	0.3	− 0.495	1.049
SCAS-P^b^										
n	15	14				15	12			
Total	47.73 (18.10)	44.64 (21.91)	0.2	− 0.0575	0.883	51.53 (17.01)	39.17 (20.37)	0.7	− 0.116	1.433
SCAS-C^b^										
n	15	14				13	13			
Total	46.87 (15.16)	37.71 (18.02)	0.5	− 0.191	1.292	46.15 (19.78)	33.85 (14.31)	0.7	− 0.08	1.505
CAPE^c^										
n	14	14				13	13			
Formal activities: diversity	4.57 (3.37)	4.07 (2.53)	0.2	− 0.0574	0.91	4.15 (2.48)	4.23 (1. 83)	0.04	− 0.806	0.732
Intensity	1.55 (1.17)	1.39 (0.84)	0.2	− 0.585	0.899	1.43 (1.12)	1.40 (0.71)	0.03	− 0.737	0.801
Informal activities: diversity	22.50 (4.67)	22.29 (6.28)	0.04	− 0.703	0.779	21.38 (4.46)	23.00 (3.94)	− 0.4	− 1.161	0.391
Intensity	2.86 (0.63)	3.15 (0.97)	− 0.3	− 1.101	0.392	2.67 (0.71)	2.85 (0.68)	− 0.3	− 1.031	0.513

^a^FSSC-R Fear Survey Schedule for Children—Revised, higher scores indicate greater anxiety severity

^b^Spence Children’s Anxiety Scale—parent version (SCAS-P) and child version (SCAS-C)

^c^CAPE—Children’s Assessment of Participation and Enjoyment

Data from 2 weeks and 6 months after the control group received treatment are presented in Table 7. In combination, the post treatment data for the immediate treatment and control groups showed 13/31 (41.9%) children were treatment responders at 2 weeks post treatment, and 11/24 (45.8%) children at 6 months post treatment.

**Table 7:** Target behaviour ratings categories (responder, no change/equivocal and worse) for delayed treatment control group at 2 weeks and 6 months after receiving treatment

Target behaviour rating	Control group post treatment	
	2 weeks (n = 15)	6 months (n = 8)
	n (%)	n (%)
Treatment responder (1.0–3.0)	9 (60.0)	5 (62.5)
No change/equivocal (3.1–5.9)	5 (33.3)	3 (37.5)
Symptoms worse than at baseline (6.0–9.0)	1 (6.7)	0

## Discussion

This is the first report of a randomised controlled trial of an immersive virtual reality treatment for specific phobia in young people with ASD.

The trial results regarding treatment fidelity and trial retention support our previous findings of the feasibility and acceptability of this novel intervention for specific phobia (Maskey et al. 2014). NHS clinicians were keen to refer; families were positive about accessing treatment through a trial including randomisation. All children and parents in the immediate treatment group attended four sessions of treatment. Treatment was delivered with fidelity by 11 assistant psychologists and specialist nurses, after a 2 hours training workshop and initial supervision. During treatment, most children’s rating of their confidence at tackling their goal situation increased, an increase reflected in parent ratings. Data completeness for the main outcome measure of target behaviour rating was excellent.

One-third of children from the treatment group showed improvements in their real life targeted phobia, with children able to manage everyday activities and situations that were not possible previously. By contrast, no children in the control group showed improvement in their specific phobia during their wait phase of the trial period. Furthermore, five control group children showed a clear deterioration in target behaviour rating from baseline, compared with one treatment group child. When the control group later received treatment, a similar proportion were classified as responders as to the immediate treatment group.

More than half the children in the immediate treatment group were not rated as ‘responders’. Whilst a success rate of 38% with definite positive change on blinded, independent rating, with a large effect size, is at least comparable with other interventions for anxiety (Ung et al. 2015), we will explore in a future report factors that may moderate responsiveness to the intervention.

Regarding the questionnaire measures, the descriptive data indicates that these anxiety tools developed for use with typically developing individuals were not sensitive to the treatment effect observed in the main target behaviour outcome which focused on individualised real life examples. While collection of data using these questionnaires seemed acceptable to young people and parents, we will assess their usefulness as outcome measures in future studies. In line with other research investigating treatment of fears and phobias in ASD (Lydon et al. 2015), we conclude that target behaviour ratings are the best indicator of real life change and will be used as we continue to monitor the effectiveness of the treatment. Behaviour vignettes are straightforward to collect from parents (by brief telephone interview), can be gathered ‘blind’, and are acceptable as indicated by the high level of data completeness.

This is a methodologically strong single blind trial, including randomisation, delivery of a manualised treatment with high fidelity levels, 100% retention, comprehensive outcome measurement conducted by a blinded assessor, and target behaviour ratings evaluated by an independent blinded panel. There were possible weaknesses, including the sample size, and potential for placebo effect in self-report. Updates on participants’ functioning were obtained by parent report, which has the potential for bias; direct observations of progress were not made. Due to the need to accommodate scene design and parent availability, there were small differences between the time from baseline measurement to treatment, and treatment to outcome measurement—although these were the same for the treatment and control groups.

### Clinical Implications and Future Research

New interventions for anxiety in children with ASD are needed. Whilst CBT can be effective, adaptations to CBT have been shown to improve the efficacy of this form of therapy for autistic people (Lang et al. 2010; Moree and Davis 2010; Sofronoff et al. 2005; Weiss et al. 2018). The Blue Room VRE treatment conforms with guidance contained in the UK National Institute for Health and Care Excellence (NICE) in Guideline 170 (NICE 2013) regarding adaptations to CBT for children with ASD. Adaptations included in this treatment included greater use of written and visual information, a more cognitively concrete and structured approach, and involving a parent/carer. Additional reasons why virtual reality can improve the effectiveness of CBT for children with ASD include being able to repeatedly practice anxiety reduction techniques at one level of exposure to enable mastery to be achieved before moving up the exposure hierarchy. This develops confidence and a feeling of control as the child receives controlled exposure. Parents observe sessions and gain an understanding of the methods used, which they can utilise in real life situations. Virtual reality is increasingly being used in mental health therapy with positive effect in the neurotypical population (Maples-Keller et al. 2017; Botella et al. 2017; Parsons and Rizzo 2008); the results from the study presented here illustrate that this type of intervention may also be beneficial for people with autism.

In future research, comparing treatment with a group receiving traditional CBT only would more directly evaluate the additional impact of the VR component. The feasibility and data requirements of a cost-effectiveness analysis are being assessed. Investigating what adaptations are required for the treatment of people with mild or moderate learning disability will be important. Characteristics of children and families who benefit most from treatment will be studied as the treatment is delivered through clinical services, or future studies. As clinical delivery of the Newcastle Blue Room VRE Treatment progresses, systematic data gathering with all children and families is planned.
